# Magnetic moments induce strong phonon renormalization in FeSi

**DOI:** 10.1038/ncomms9961

**Published:** 2015-11-27

**Authors:** S. Krannich, Y. Sidis, D. Lamago, R. Heid, J.-M. Mignot, H. v. Löhneysen, A. Ivanov, P. Steffens, T. Keller, L. Wang, E. Goering, F. Weber

**Affiliations:** 1 Institute for Solid State Physics, Karlsruhe Institute of Technology, 76021 Karlsruhe, Germany; 2 Laboratoire Léon Brillouin (CEA-CNRS), CEA Saclay, F-91911 Gif-sur-Yvette, France; 3 Physics Institute, Karlsruhe Institute of Technology, 76049 Karlsruhe, Germany; 4 Institute Laue-Langevin, BP 156, 38042 Grenoble, France; 5 Max Planck Institute for Solid State Research, D-70569 Stuttgart, Germany; 6 Max Planck Society, Outstation at the FRM II, D-85748 Garching, Germany; 7 Max Planck Institute for Intelligent Systems, 70569 Stuttgart, Germany

## Abstract

The interactions of electronic, spin and lattice degrees of freedom in solids result in complex phase diagrams, new emergent phenomena and technical applications. While electron–phonon coupling is well understood, and interactions between spin and electronic excitations are intensely investigated, only little is known about the dynamic interactions between spin and lattice excitations. Noncentrosymmetric FeSi is known to undergo with increasing temperature a crossover from insulating to metallic behaviour with concomitant magnetic fluctuations, and exhibits strongly temperature-dependent phonon energies. Here we show by detailed inelastic neutron-scattering measurements and *ab initio* calculations that the phonon renormalization in FeSi is linked to its unconventional magnetic properties. Electronic states mediating conventional electron–phonon coupling are only activated in the presence of strong magnetic fluctuations. Furthermore, phonons entailing strongly varying Fe–Fe distances are damped via dynamic coupling to the temperature-induced magnetic moments, highlighting FeSi as a material with direct spin–phonon coupling and multiple interaction paths.

## Abstract (web summary)

Whilst emergent phenomena and potential technological applications in materials may rely of the coupling between spin, lattice, and electronic degrees of freedom, little is known about direct coupling mechanism between spins and phonons. Here, the authors evidence such behaviour in noncentrosymmetrc FeSi.

## Graphical abstract

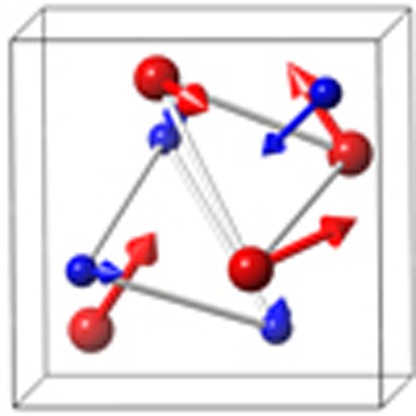

The family of transition metal silicides crystallizing in the noncentrosymmetric cubic B20 structure exhibits a variety of complex phenomena, which are of interest to both basic and applied science^1^,^2^,^3^. In MnSi, the helical arrangement of Mn spins results in exotic phases such as partial order under hydrostatic pressure^4^ or a skyrmion lattice in an applied magnetic field^5^. The latter is also found in Fe_1−*x*_Co_*x*_Si (ref. 6). Pure FeSi has been under intense research for over 40 years owing to its puzzling non-magnetic ground state^7^,^8^ and recent experiments opened the perspective for potential applications in spintronics^9^ and thermoelectricity^10^,^11^.

FeSi, a semiconductor with a small bandgap Δ of 60–70 meV (refs 7, 8, 12), shows similarities to the class of heavy-fermion Kondo insulators. Its foremost peculiarity is a large temperature-induced paramagnetic moment of >2 μ_*B*_, while the ground state is non-magnetic^7^,^13^. The mechanism responsible for the temperature-activated magnetization and the strong temperature dependence of the bandgap in FeSi has remained controversial ever since its discovery back in the 1960s (refs 7, 8). Inelastic polarized neutron scattering showed a sharp increase with increasing temperature of magnetic scattering close to reciprocal lattice points^14^,^15^, indicating the existence of ferromagnetic correlations^16^. Recently, thermal lattice disorder^17^, as well as strong electron–electron correlations^18^, has been invoked to explain the unusual properties of FeSi. In particular, the strong reduction of the gap Δ on heating towards room temperature^12^ is observed in an unexpectedly low-temperature range with respect to the gap value of 60–70 meV.

Although the average atomic structure of FeSi does not exhibit any notable changes with temperature, anomalous phonon properties have been reported recently from Raman^12^ and inelastic neutron-scattering measurements^17^. The authors of those publications found a surprisingly strong softening of phonon energies with increasing temperature, which they related to the closing of the charge gap in FeSi. However, the information obtained was limited because of restrictions in the accessible range of temperature and wave vector of Raman measurements. Furthermore, most inelastic neutron-scattering data were obtained on powder samples, not allowing an investigation of the lifetimes of phonon modes, or their directional dependences in reciprocal space.

We have performed inelastic neutron-scattering experiments on a high-quality single crystal of FeSi. This allows us to determine phonon dispersion curves. In addition, we obtain detailed information on the temperature dependence of the phonon linewidths that are directly linked to the lifetimes of lattice vibrations, across the transition from a non-magnetic semiconductor to a metal with a large paramagnetic moment. Large temperature-dependent intrinsic linewidths have been observed for several phonon modes, which simultaneously show decreasing coherent intensities and very strongly temperature-dependent energies. We find that the temperature dependences of linewidths and intensities agree well with that of the temperature-induced magnetic moment. *Ab initio* lattice dynamical calculations demonstrate that reduced phonon lifetimes can be understood only if we include the evolution of magnetism. In conjunction with this behaviour, the reduction of phonon intensities goes far beyond the quasi-harmonic approximation. We argue that the particular ionic motions associated with the investigated phonons can lead to additional renormalization as temperature increases, via a dynamical coupling to the growing magnetic moments.

## Results

**Figure 1:**
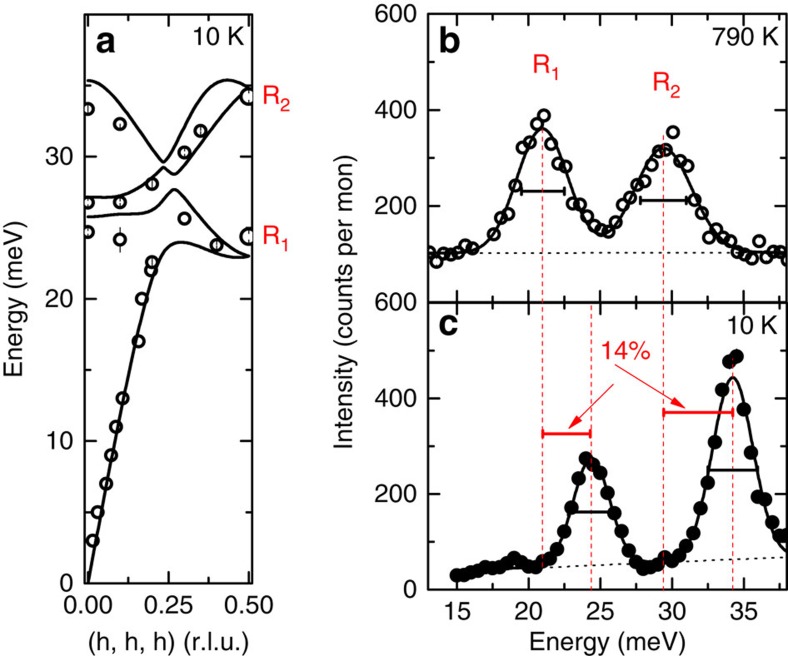
Representative inelastic neutron-scattering data and dispersion at the **R** point. (**a**) Measured and calculated phonon dispersion in FeSi from the centre of the Brillouin zone towards the **R** point, **Q**=(1.5, 1.5, 1.5), which is the zone boundary in the crystallographic [111] direction. Shown are branches with longitudinal symmetry. (**b**,**c**) Measured neutron intensities at the **R** point at temperatures (**b**) *T*=790 K and (**c**) *T*=10 K. Solid lines denote fits to the data using a Lorentzian line shape convoluted with the experimental resolution on top of the experimental background (dotted horizontal lines). Dashed (red) vertical lines indicate the observed peak energies and mark the softening of the R1 and R2 modes between *T*=10 and 790 K by 14% of the low-temperature values. Thick horizontal bars denote the calculated experimental resolution. Error bars represent s.d.

To establish an understanding of the lattice dynamical properties of FeSi in detail, we performed extensive measurements of the phonon modes along various high-symmetry directions in reciprocal space at *T*=10 K. Phonons were primarily investigated using inelastic neutron scattering on the thermal triple-axis spectrometer (TAS) 1 T at the ORPHEE reactor at Laboratoire Léon Brillouin at CEA Saclay and results were compared with *ab initio* calculations based on density functional perturbation theory (DFPT). To that end, we used the experimental low-temperature lattice constants^19^ and optimized the internal parameter of the unit cell to achieve a force-free equilibrium structure. In the following, all wave vectors are expressed in reciprocal lattice units of 2*π*/*a*, where *a*=4.48 Å is the low-temperature lattice constant of the cubic unit cell of FeSi. Overall, we find very good agreement between the observed and calculated energies (Fig. 1a; Supplementary Fig. 1).

**Figure 2:**
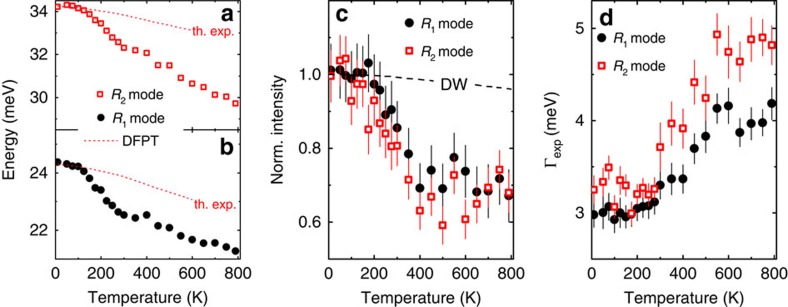
Temperature dependence of the phonon properties at the **R** point. (**a**,**b**) Temperature evolution of the energy of the (**a**) R1 and (**b**) R2 phonon modes. The dashed lines denote the calculated energies from the quasi-harmonic approximation using temperature-dependent lattice constants reported in ref. 19 and simulating the effect of thermal expansion (th. exp.). (**c**) Temperature evolution of intensities of the R1 and R2 modes corrected for the Bose factor and normalized to their values at *T*=*10* K. The dashed line indicates the expected evolution because of the temperature-dependent Debye–Waller factor [19] (DW). (**d**) Temperature dependence of the observed linewidths Γ_exp_ of the R1 and R2 modes. Error bars represent s.d.

We have studied the phonon properties over a wide range of temperature (10 K≤*T*≤790 K) at various wave vectors for which phonon selection rules are unambiguous. Note that the rather low symmetry of the B20 crystal structure typically results in unclear phonon selection rules, that is, various phonon modes contribute to the spectral weight distribution in the corresponding energy scans. Accordingly, the linewidths of the different phonon peaks can no longer be accurately determined. Raw data taken at **q**=(½, ½, ½), that is, at the Brillouin zone boundary **R** point along the [111] direction, are shown in Fig. 1b,c. We find two well-separated phonon peaks, in agreement with the DFPT predictions, at energies of 24.3 and 34.2 meV for *T*=10 K. For simplicity, we call them R1 and R2 modes, respectively, in the following. We observe a very strong softening of the phonon energies, by 14% for both modes, in the investigated temperature range, which is in good agreement with the reported temperature dependence of a peak observed previously in the phonon density of states^17^. The detailed study of the present work, however, demonstrates that the softening exhibits quite unusual temperature dependence. Over the whole temperature range between 10 and 790 K, we observe three distinct regions (Fig. 2a,b): (1) the temperature dependence is rather weak for *T*≤100 K. In our calculations, we show that in this temperature range, the softening can be quantitatively attributed to the increase in the lattice constants using values obtained by neutron diffraction^19^ (see dashed lines in Fig. 2a,b). (2) At intermediate temperatures (100 K≤*T*≤300 K), we observe a strong anomalous phonon softening, with a factor of ∼4 stronger decrease of the phonon energy than predicted based on the thermal expansion (see dashed line in Fig. 2a,b). (3) Finally, for 300 K≤*T*≤790 K, a weaker softening rate is recovered, with a decreasing energy consistent with the measured lattice expansion. We note that many other phonon modes show qualitatively similar temperature dependences, although the strength of the softening varies. There are also some modes for which the energies fully follow the expectations from thermal expansion without an additional softening. Thus, the anomalous softening is related to specific phonon symmetries. A complete report is beyond the scope of this paper and will be published in the future.

We can further analyse the temperature dependence of the phonon intensities and linewidths at wave vectors with unambiguous phonon selection rules. In particular, we can accurately determine the experimental background at the **R** point, and derive the energy-integrated intensities and the linewidths *Γ*_exp_ (full width at half maximum) in a first step from Gaussian fits to the raw data of the R1 and R2 modes. Both quantities show anomalous behaviour, again in the similar temperature range 200–400 K. After correcting for the temperature-dependent phonon intensities due to the Bose factor, we find that the energy-integrated intensities of both R1 and R2 show a rapid decrease by 30% in the range 200 K≤*T*≤400 K, and then level off towards higher *T* (Fig. 2c). At low temperature, *Γ*_exp_ agrees well with the calculated experimental resolution of 3 and 3.2 meV for the R1 and R2 modes, respectively. Thus, the intrinsic phonon linewidth Γ_phon_ cannot be resolved in this *T* range. At higher temperatures, at which *Γ*_exp_ deviates from the instrumental resolution, we can extract *Γ*_phon_ by fitting a Lorentzian function, which is typically a good approximation of the spectral function of a damped phonon as long as *Γ*_phon_ is much smaller than the mode energy *ħω*, convoluted with the Gaussian experimental resolution to the data (Fig. 1b). For *T*≥200 K, the linewidth increases strongly and levels off at 500 K (Fig. 2d). The temperature dependences are qualitatively similar for both R1 and R2, with an increase of *Γ*_exp_ by 30 and 50%, respectively. Generally, phonons exhibit a reduced intensity with increasing temperature due to thermal atomic motions, which reduce the phonon intensity according to the Debye–Waller factor^20^. Furthermore, incoherent atomic motions hamper an easy propagation of phonons through the lattice and, hence, lead to smaller phonon lifetimes, that is, larger linewidths. However, the observed strong temperature dependences cannot be explained by these effects. First, the observed temperature dependence of the Debye–Waller factor^19^ only accounts for an intensity loss by about 4% (see dashed line in Fig. 2c) in the temperature range investigated. Second, the temperature dependence of the Debye–Waller factor is smooth, in stark contrast to our observations^19^.

**Figure 3:**
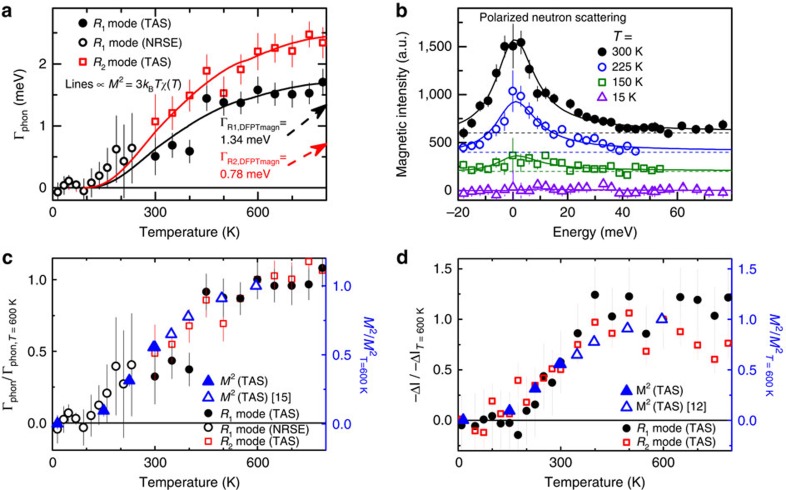
Temperature dependences of lattice and magnetic degrees of freedom in FeSi. (**a**) Phonon linewidths Γ_phon_ (full width at half maximum) of the R1 and R2 modes obtained from TAS (*T*≥*300* K) and neutron resonant spin-echo (NRSE; *T≤230* K) measurements. Solid lines represent 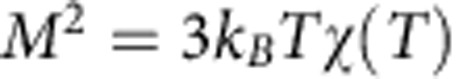 based on our magnetization measurements and scaled to fit the high temperature values of Γ_phon_. Dashed arrows indicate the predicted linewidths for the R1 and R2 modes in spin-polarized DFPT calculations. (**b**) Paramagnetic scattering measured at 15 K≤T≤300 K employing the differential method. Data are vertically offset for clarity. Colour-coded dashed lines denote the respective zero lines. Solid lines are fits using equation (1). (**c**) Comparison of the temperature dependence of Γ_phon_ of the R1 and R2 modes with that of the temperature-induced paramagnetic moment *M*^2^(*T*) extracted from polarized neutron scattering (see **b**). Values of *M*^2^(*T*) at *T*≥300 K were taken from ref. 15 and scaled using the value at *T*=300 K. All data are normalized to their respective value at *T*=600 K. (**d**) Comparison of the reduction of the intensities of the R1 and R2 modes with increasing temperature with *M*^2^(*T*) derived from polarized neutron scattering. Again, all data are normalized to their respective value at *T*=600 K. Error bars represent s.d.

As already mentioned, we can observe finite values of *Γ*_phon_ for the two phonon modes R1 and R2 only for *T*≥300 K because statistical error bars on *Γ*_exp_ are about ±(0.15–0.25) meV due to the relatively low experimental resolution of the TAS. To investigate the evolution of *Γ*_phon_ at lower temperatures, we performed neutron resonant spin-echo measurements on the polarized thermal neutron TRISP spectrometer at the Heinz-Maier-Leibnitz Neutron Research Facility (FRM-II) in Garching. In this technique, the precession of the neutron spin in magnetic fields before and after being scattered in the sample is used to achieve very high energy resolution^21^,^22^,^23^. The resulting temperature dependence of *Γ*_phon_ for the R1 mode in the range 15 K≤*T*≤230 K is shown in Fig. 3a along with results from the TAS measurements for *T*≥300 K. In the former, a constant *Γ*_0_=0.35 meV, which we assign to anharmonic interactions and crystal imperfections, was subtracted from the neutron resonant spin-echo results. We find that the increase of *Γ*_phon_ starts just above *T*=100 K, that is, well below room temperature, in agreement with the onset of the strong softening and levels off at high temperatures.

In the following, we will show that the observed phonon anomalies can be related to the magnetism in FeSi, which has long been known to exhibit an unusual temperature dependence on the same *T* region.

We investigated the magnetic degrees of freedom via polarized inelastic neutron-scattering and magnetization measurements. Results from the latter are shown in Supplementary Fig. 2 and agree well with previous reports^7^,^8^. In Fig. 3a, we plot the (scaled) square of the temperature-induced magnetic moment *M*^2^(*T*) calculated via *M*^2^(*T*)=3*k*_B_*T* × *χ*(*T*) and compare it with Γ_phon_(*T*) of the R1 and R2 phonon modes. We find good agreement between the increase of *M*^2^(*T*) and Γ_phon_(*T*), indeed indicating a close relationship between magnetism and the lattice dynamical properties in FeSi. Pramagnetic scattering obtained via polarized inelastic neutron scattering close to the (1, 1, 0) Bragg position is shown in Fig. 3b (Supplementary Fig. 3; Supplementary Note 1). The data demonstrate that magnetic fluctuations present at room temperature vanish completely at a temperature between 15 and 150 K. In agreement with previous reports^14^,^15^, the observed scattering can be described by the paramagnetic scattering function


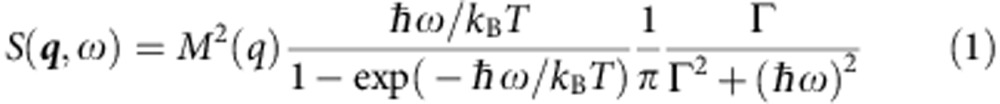


using a single value for the energy width Γ_magn_=(10±1) meV of the paramagnetic scattering at all temperatures (Fig. 3b). Hence, the increase of magnetic scattering with temperature is qualitatively the same at all energies.

We compare Γ_phon_(*T*) of the R1 and R2 modes with *M*^2^(*T*) extracted from polarized neutron scattering (Fig. 3c) and with the intensity reduction of both phonon modes (Fig. 3d). Here we include results presented in ref. 15 for *M*^2^(*T*) at *T*≥300 K. The absolute values were scaled to match our result at room temperature.

We find good agreement in both comparisons demonstrating that also the intensity reduction is linked to the evolution of magnetism in FeSi. At this point, we emphasize that neutron diffraction data do not reveal any significant magneto–elastic coupling, that is, the lattice constant increases smoothly on heating^19^. Hence, any coupling between phonons and magnetism must be dynamic in origin.

We now return to the *ab initio* lattice dynamical calculations that yield a smooth evolution of the phonon energies R1 and R2 when taking only the thermal lattice expansion into account (Supplementary Note 2). Besides phonon energies, we can also calculate the electronic contribution to the phonon linewidth *γ*, that is, the coupling strength of a specific phonon mode to the electronic states. Because of the charge gap, there is no Fermi surface and, hence, no electron–phonon coupling (EPC) in FeSi at low temperatures. Indeed, our electronic structure calculations show a strong suppression of the electronic density of states near *E*_F_, which is reminiscent of a gap (Supplementary Fig. 4). The charge gap in FeSi decreases strongly on heating towards room temperature^12^ and hence EPC can be present in the metallic regime. However, calculations performed for metallic FeSi (Supplementary Note 2) yield linewidths smaller than 0.1 meV for all phonons, much smaller than the observed values (Fig. 3a). We conclude that a mere metallization of FeSi cannot explain our observations.

Motivated by the very similar temperature evolution of Γ_phon_(*T*) and *M*^2^(*T*), we have performed spin-polarized calculations. Although DFPT, in agreement with experiment, does not yield a stable magnetically ordered ground state for FeSi, we found that such a state can be stabilized by increasing the lattice constant to a*=4.65 Å, that is, by nearly 4% with respect to the experimental lattice constant. Spin-polarized calculations with a*=4.65 Å yield a ferromagnetically ordered ground state with an ordered moment of 0.8 *μ*_B_ per Fe atom (Supplementary Fig. 4). Although FeSi at elevated temperatures is a paramagnet, the assumption of ferromagnetic short-range correlations is not unreasonable, the scattering due to magnetic fluctuations strongly peaks on approaching reciprocal lattice vectors with large ferromagnetic structure factor^15^, which is a clear signature of short-range ferromagnetic correlations (Supplementary Fig. 3; Supplementary Note 1)^16^. Indeed, properties of FeSi have been interpreted in terms of a nearly ferromagnetic semiconductor^24^. Finally, closely related compounds such as FeGe show helical magnetic order with a small pitch between neighbouring Fe moments, which on short-length scales resembles ferromagnetic order^25^ (Supplementary Note 2).

**Figure 4:**
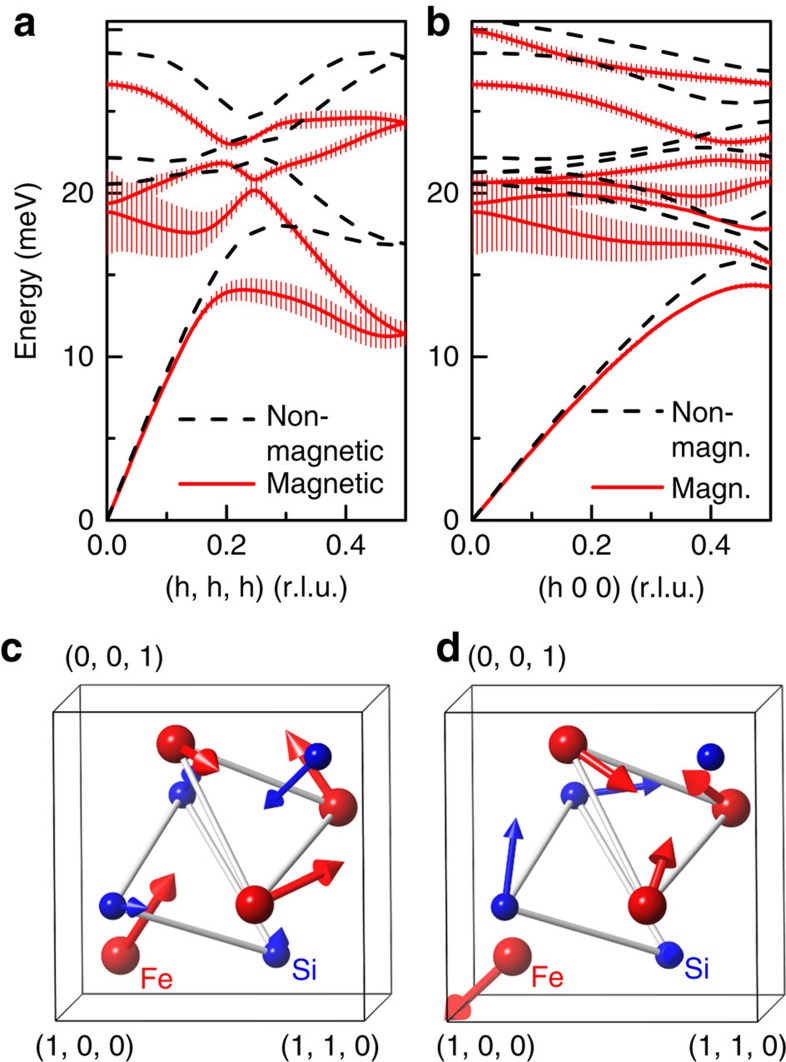
Results from spin-polarized DFPT calculations. (**a**,**b**) Calculated phonon dispersions using a*=4.655 Å with (solid lines) and without (dashed lines) ferromagnetic spin polarization along the (**a**) [111] and (**b**) [100] directions. For clarity, only branches with longitudinal symmetry are shown. The calculated electronic contribution *γ* to the phonon linewidth is represented as error bars in the data of the spin-polarized calculation. In the non-magnetic calculation, results for *γ* are <0.1 meV for all phonon modes. (**c**,**d**) Unit cell of FeSi with the calculated eigenvector of the (**c**) R1 and (**d**) R2 modes indicated by arrows (qualitative). Coordinates in real space are given in lattice constants units (*a, a, a*). r.l.u., reciprocal lattice unit.

Results for the lattice dynamics for non-magnetic and ferromagnetic FeSi, both with a*=4.65 Å, are shown for two high-symmetry directions in Fig. 4a,b. Compared with the dispersion presented in Fig. 1a, energies are generally softer as a result of the increased lattice constant. The non-magnetic calculations with different lattice constants show different phonon energies by about 25% (as can be seen by comparing Fig. 4a (a*=4.65 Å) and Fig. 1a (*a*=4.48 Å)). Importantly, introducing magnetism in FeSi with the same lattice constant a* causes additional softening by up to 10%, which is observed at the Brillouin zone centre and over the whole wave vector range, for example, along the [100] direction (Fig. 4b). The magnetism-induced effect on phonons is particularly strong for the R1 mode, with a softening of 35%, and still relatively large for the R2 mode (15%). Furthermore, the spin-polarized calculation predicts strong overall EPC, with an EPC constant of *λ*≈1, which is similar to values obtained for conventional superconductors with medium to strong EPC^26^,^27^.

Accordingly, several phonon branches, in particular the R1 and R2 modes, are strongly damped and exhibit large values of the calculated linewidth *γ* due to EPC (Fig. 4a,b). We were able to investigate two more phonon modes, one at **q**=(0.25, 0.25, 0) and the other at the zone centre. Spin-polarized DFPT predicts essentially zero EPC for the former mode but very strong EPC for the latter. Both predictions are verified by our measurements (Supplementary Fig. 5; Supplementary Note 3). Our analysis therefore indicates that temperature-induced magnetism is directly responsible for the increasing phonon linewidths, which can account for the close similarity between Γ_phon_(*T*) and *M*^2^(*T*). We note that our calculations do not include a direct spin–phonon coupling (SPC). Rather, the large linewidths in spin-polarized DFPT originate from a change in the electronic states at the Fermi surface, which are mediating EPC.

## Discussion

On the other hand, our calculations underestimate the absolute value of the linewidths by 30% for the R1 mode and even a factor of three for the R2 mode and do not predict the intensity reduction seen in Fig. 2c. Large phonon linewidths, for which DFPT correctly predicted the wave vector dependence but underestimated the absolute strength, have also been found in some charge density wave compounds^28^,^29^ and superconductors^26^. The likely origin of the discrepancies, in those cases, is that DFPT does not include anharmonic effects, which definitely play an important role in charge density wave materials. For FeSi, an explanation might include a direct SPC. The impact of SPC on the phonon lifetime in a Heisenberg ferromagnet has been calculated, where the lowest-order contribution to the phonon lifetime originates from processes, in which the phonon emits and later reabsorbs two magnons^30^. In an ordered ferromagnet, the strength of the renormalization depends sensitively on the overlap of the wave vector and energy position of the renormalized phonon with those of a bound state of the two emitted spin waves. In contrast to well-defined spin waves, magnetic intensities in FeSi show qualitatively the same temperature dependences independent of their respective energy (Fig. 3b). Hence, one would expect the same temperature dependences for phonon renormalization effects due to SPC independent of phonons' respective energies. Thus, this scenario of phonons coupling to magnetic excitations naturally explains the concomitant increase of magnetic scattering and phonon renormalization in FeSi (Fig. 3c).

Our results show that there are also phonons, which do not exhibit strong renormalization. Therefore, it is interesting to investigate the phonon displacement patterns to look for common features in modes with strong renormalizations and differences with regard to phonon modes showing no response to the growing magnetism. We have computed the atomic displacement patterns for the R1 and R2 modes (Fig. 4c,d), as well as for the other two modes discussed above (Supplementary Fig. 5) from our DFPT calculations. Indeed, we find that Fe–Fe distances do strongly change for the modes exhibiting large linewidths at high temperatures, whereas the pattern of the mode at **q=**(0.25, 0.25, 0) with zero EPC features motions of the Fe atoms in the unit cell with much smaller changes in the Fe–Fe distances. This difference directly reflects the strong interaction between magnetic exchange that decisively depends on the Fe–Fe distance and the lattice degrees of freedom. Another interesting scenario considers the coupling between the time-dependent electric polarization induced by vibrating ions and the magnetic moment of the Fe 3*d* electrons. Although, this situation has—to our knowledge—not yet been considered theoretically, we would also qualitatively expect that phonons, whose eigenvectors exhibit strong changes in the Fe–Fe distances, show the strongest effects. Hence, FeSi appears to be a rare example of SPC (in contrast to the rather common effects on phonons because of magneto–elastic coupling). An increasing anharmonicity due to SPC could explain the reduction of the phonon intensities that is also similar to the temperature dependence of *M*^2^(*T*) (Fig. 3d).

In conclusion, we have reported temperature-dependent phonon renormalization in FeSi over a wide temperature range. Our analysis using DFPT calculations demonstrates that our observations are directly related to the temperature-induced magnetism. Going beyond the quasi-harmonic approximation, our results suggest that FeSi exhibits a purely dynamic SPC, which enhances the renormalization predicted by DFPT. Therefore, FeSi is not only a very peculiar paramagnet but may serve as a model compound for strongly interacting magnetic and lattice degrees of freedom.

## Methods

### Sample characterization

Our single crystal of FeSi weighing 35 g was characterized in terms of magnetization and electrical resistivity, as well as Larmor diffraction and crystal mosaicity using neutrons (for results see Supplementary Fig. 2). Magnetization measurements have been performed by a new Quantum Design MPMS3 system, utilizing the 1,000-K oven option above 300 K. The measurements have been performed in vibrating sample magnetometer (VSM) mode with 1-mm peak-to-peak amplitude. The VSM data have been subsequently normalized to room temperature d.c. mode results, to obtain high-quality absolute magnetization values. No further data processing has been performed. The sample was a piece cut from the large single crystal used for neutron scattering with *m*=0.033 g and dimensions (4 × 2 × 1) mm^3^. Standard Four-Point Probe resistivity measurement has been performed using the physical properties measurements system (PPMS) a.c. transport function to control the temperature from 5 to 300 K and excitation current to be 0.1 mA. The sample cut from the large single crystal used for neutron scattering had the dimensions (4 × 1 × 1) mm^3^. The contacts are made by connecting 20-μm Ag wires using conductive silver paint. The distance between the two leads for the voltage measurement was 3.5 mm. Therefore, we calculated the resistivity, 
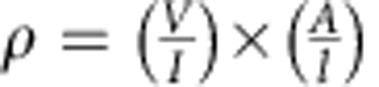
 (*V*: voltage; *I*: excitation current; *A*: area, that is, 1 × 1 mm^2^; *l*: distance between leads).

### Inelastic neutron scattering

Inelastic neutron scattering was used to study the phonons on the thermal TAS 1T at the ORPHEE reactor at Laboratoire Léon Brillouin, CEA Saclay. Double-focusing graphite monochromator and analyser were used for all phonon measurement. The final energy at the analyser was set to 14.7 meV allowing the use of a graphite filter to suppress higher-order scattering. The single crystal was mounted in a closed-cycle refrigerator allowing measurements in the temperature range 5 K≤*T*≤790 K. The paramagnetic scattering in FeSi was studied with polarized thermal neutrons on the TAS IN20 at ILL, Grenoble, using the differential method^31^ with a standard set-up employing Heusler monochromator and analyser and a final energy of 14.7 meV. The intrinsic phonon linewidths Γ_phon_ of the R1 mode at 15 K≤*T*≤225 K were investigated using the neutron resonance spin-echo technique on the polarized thermal TAS TRISP at the neutron research source Hans-Maier Leibnitz (FRM-II) in Garching^21^,^22^ (for details of the measurement see Supplementary Note 4 and Supplementary Fig. 6). The same instrument was used to perform Larmor diffraction measurements^2^.

### Density functional theory

Density functional theory calculations were performed in the framework of the mixed basis pseudopotential method (Meyer, B., Elsässer, C., Lechermann, F. & Fähnle, M. *FORTRAN90 Program for Mixed-Basis Pseudopotential Calculations for Crystals* (Max-Planck-Institut fur Metallforschung, Stuttgart)). The exchange-correlation functional was treated in the local density approximation in the Perdew–Wang parameterization. Norm-conserving pseudopotentials for Fe and Si were constructed including the Fe 3*s* and 3*p* semicore states in the valence space. The deep potential can be efficiently treated in the mixed-basis scheme, which combines local functions together with plane waves for the representation of the valence states. Local functions of *s*, *p* and *d* symmetry at the Fe sites were combined with plane waves up to 22 Ry. Phonon frequencies and EPC were calculated using the linear response technique or DFPT^32^ in combination with the mixed-basis pseudopotential method^33^. Brillouin zone integrations were performed with a cubic 8 × 8 × 8 k-point mesh (24 points in the irreducible Brillouin zone) in combination with a standard smearing technique using a Gaussian broadening of *σ*=50 meV. Further calculations were performed using *σ*=200 meV and including spin polarization.

## Additional information

**How to cite this article:** Krannich, S. *et al.* Magnetic moments induce strong phonon renormalization in FeSi. *Nat. Commun.* 6:8961 doi: 10.1038/ncomms9961 (2015).

## Supplementary Material

Supplementary Information Supplementary Figures 1-6, Supplementary Notes 1-4 and Supplementary References.
