# Supplementary material for: Magnetic moments induce strong phonon renormalization in FeSi
Source: Nat Commun. 2015 Nov 27;6:8961. doi: 10.1038/ncomms9961 (PMC4674778; doi:10.1038/ncomms9961)
Supplement: Supplementary Information — Supplementary Figures 1-6, Supplementary Notes 1-4 and Supplementary References. [file ncomms9961-s1.pdf]

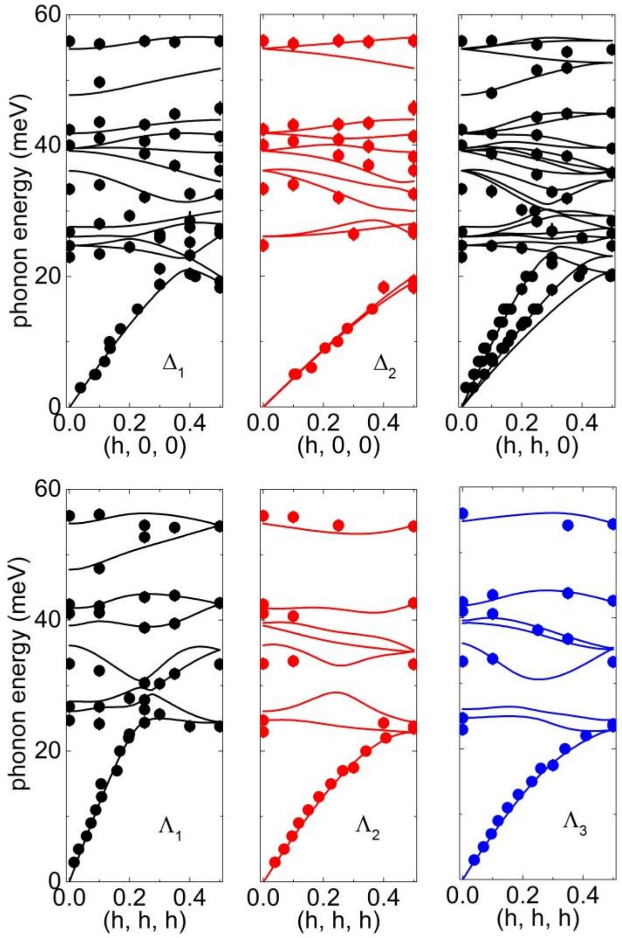

Supplementary Figure 1: Low temperature phonon dispersion of FeSi. Comparison of measured (dots) and calculated phonon energies (lines) along different high symmetry directions in FeSi at T = 10 K. If possible, results for different phonon symmetries are shown in separate panels and the respective symmetries are indicated in the panels. The DFPT calculations using the low-temperature experimental lattice constants and a Gaussian smearing of $\sigma = 0.05$ eV give a very good description of the experimental results and, hence, verify the accuracy of our ab-initio calculations. We further note that we generally found good agreement between the predicted and observed phonon intensities at specific wave vectors. Hence, DFPT gives a good description not only of the phonon energies but also the phonon eigenvectors, which define the intensity of the mode in different Brillouin zones.

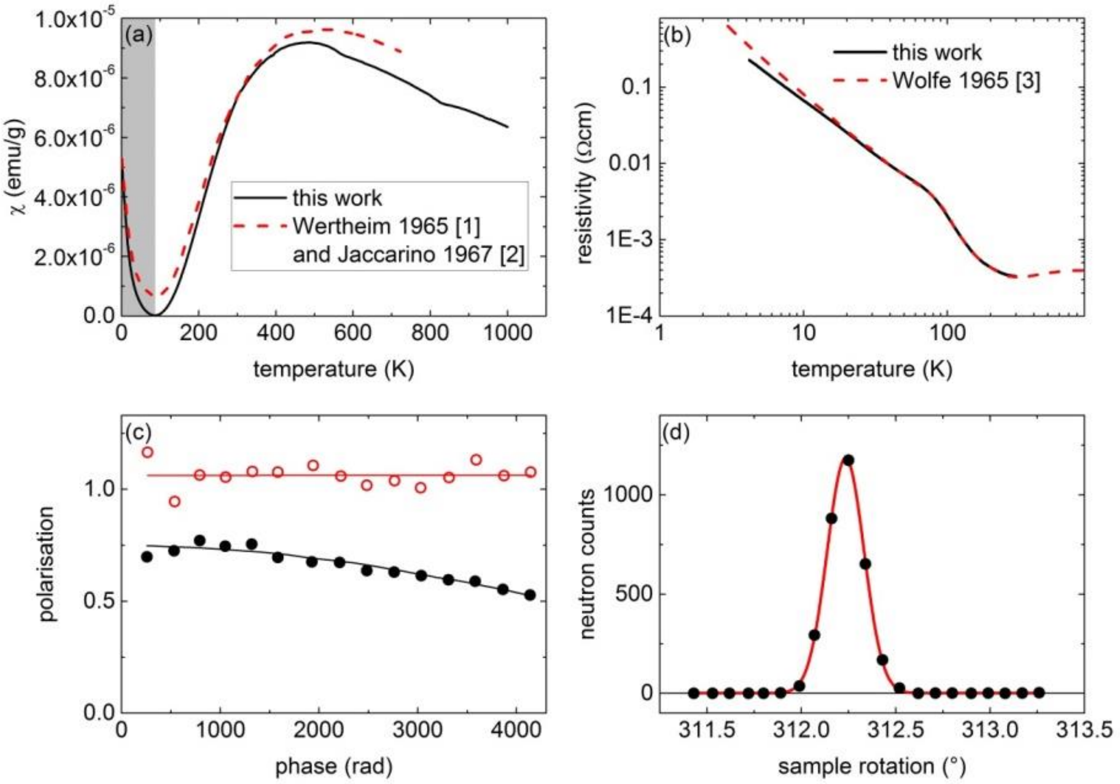

Supplementary Figure 2: Sample characterization in terms of magnetization, electrical resistivity, neutron Larmor diffraction and neutron rocking scans. *(a)* Magnetic susceptibility χ measured on a small piece of our FeSi sample (solid line). The dashed line denotes results from Refs. [1,2], which were scaled to fit our data at T = 300 K. Following the argumentation of Wertheim et al.[1], we assign the upturn of χ below $T = 90$ K (grey shaded area) to lattice defects, e.g. regions of excess Fe, and do not consider it in our analysis of the magnetic properties of FeSi. *(b)* Electrical resistivity measured on another small piece of our FeSi sample (solid line). The dashed line denotes results from Ref. [3], which were scaled to fit our data at T = 300 K. *(c)* Larmor diffraction on the (2,2,2) Bragg reflection: Data are measured in the parallel field mode, which is sensitive to the spread of d-spacings Δd and insensitive to the mosaicity. Raw data are shown in blue, whereas green data show the results normalized to the resolution P0(phase) measured with a (perfect) Ge crystal. The green curve is flat, which means that we see no broadening. The fit gives $0 \pm 4 \cdot 10^{-5}$. This error bar is reasonable, such that the spread $\Delta d_{222}/d_{222} < 4 \cdot 10^{-5}$. *(d)* Rocking scan of the (2,0,0) Bragg reflection on the 1T TAS. The scan is resolution limited, i.e. the mosaic η_{FWHM} is smaller than 0.1°.

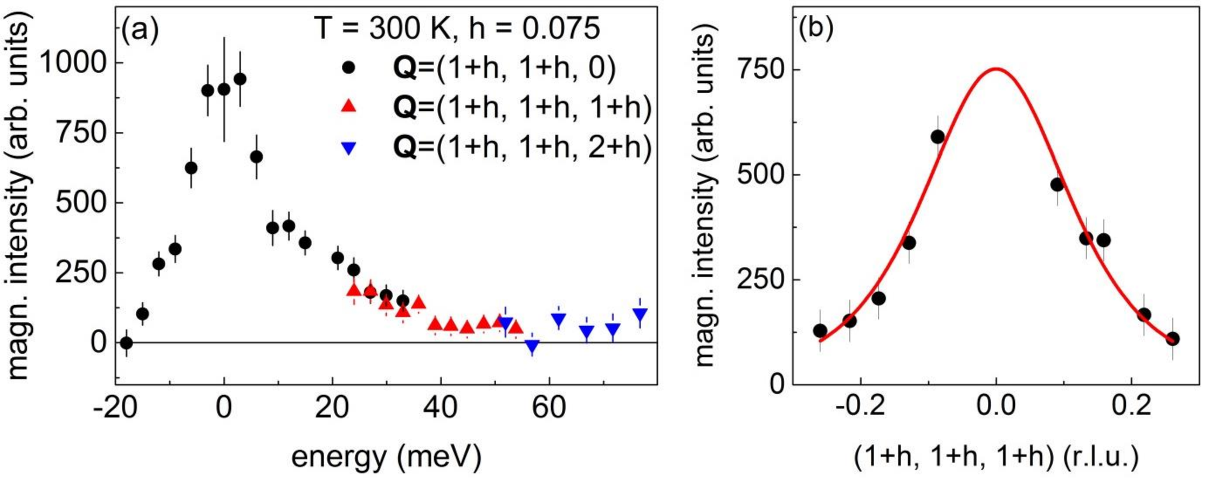

Supplementary Figure 3: Paramagnetic scattering and ferromagnetic fluctuations in FeSi. (a) Magnetic scattering obtained with polarized neutron scattering at $T = 300$ K. Measurements were performed at three different wave vectors $\mathbf{Q} = \boldsymbol{\tau} + (0.075, 0.075, 0.075)$, and $\boldsymbol{\tau}$ being a reciprocal lattice point with a large ferromagnetic structure factor (see text). (b) Amplitude of paramagnetic scattering at zero energy transfer around the reciprocal lattice vector $\boldsymbol{\tau} = (1, 1, 1)$ and T = 300 K (extracted from Ref. [4,5]). The solid line represents a fit of the data using $S(q, 0) = \frac{k_B T}{\hbar} \cdot \frac{\gamma^{-1}\chi_0}{(1+(q\xi)^2)^2}$ yielding a magnetic correlation length of $\xi = 3.6$ Å.

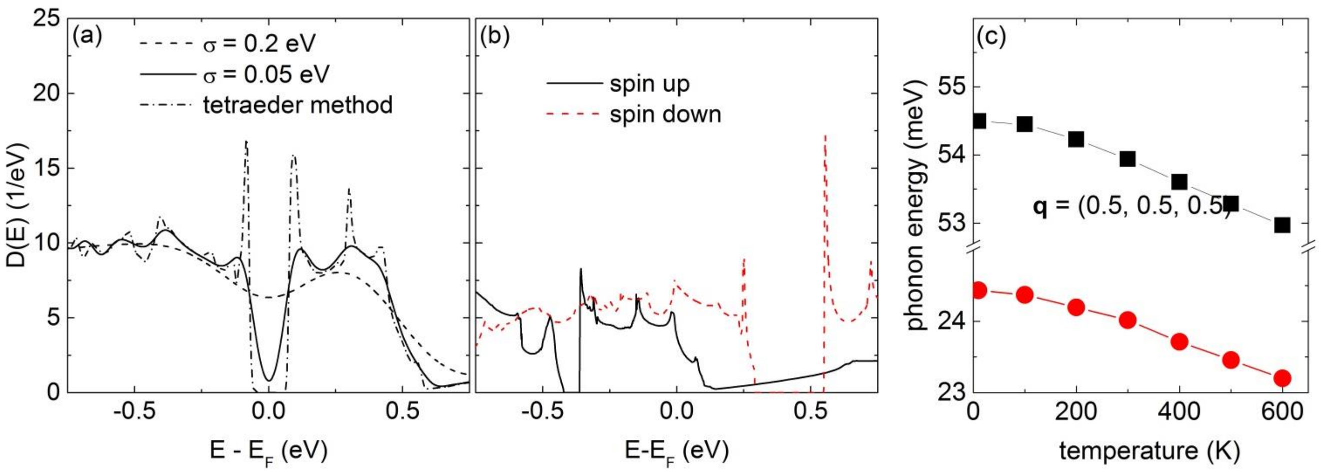

Supplementary Figure 4: Calculated electronic density of states and quasiharmonic phonon energies. *(a)* Electronic density of states (eDOS) *D(E)* in the vicinity of the Fermi energy E_F calculated within density-functional-theory (DFT) using a Gaussian smearing of σ = 0.05 eV (solid line) and 0.2 eV (dashed line). *D(E)* calculated in the more precise tetraeder method (which cannot be used in combination with phonon calculations) is shown as well (dash-dotted line). *(b) D(E)* in the vicinity of the Fermi energy E_F calculated within spin-polarized DFT in the tetraeder method. The ordered magnetic moment of the ground state is $0.8\mu_B$ per Fe atom. *(c)* Calculated frequencies of two modes at the R point using temperature dependent lattice constants taken from Ref.[6]. Results are plotted as function of the temperatures at which the lattice constants were observed.

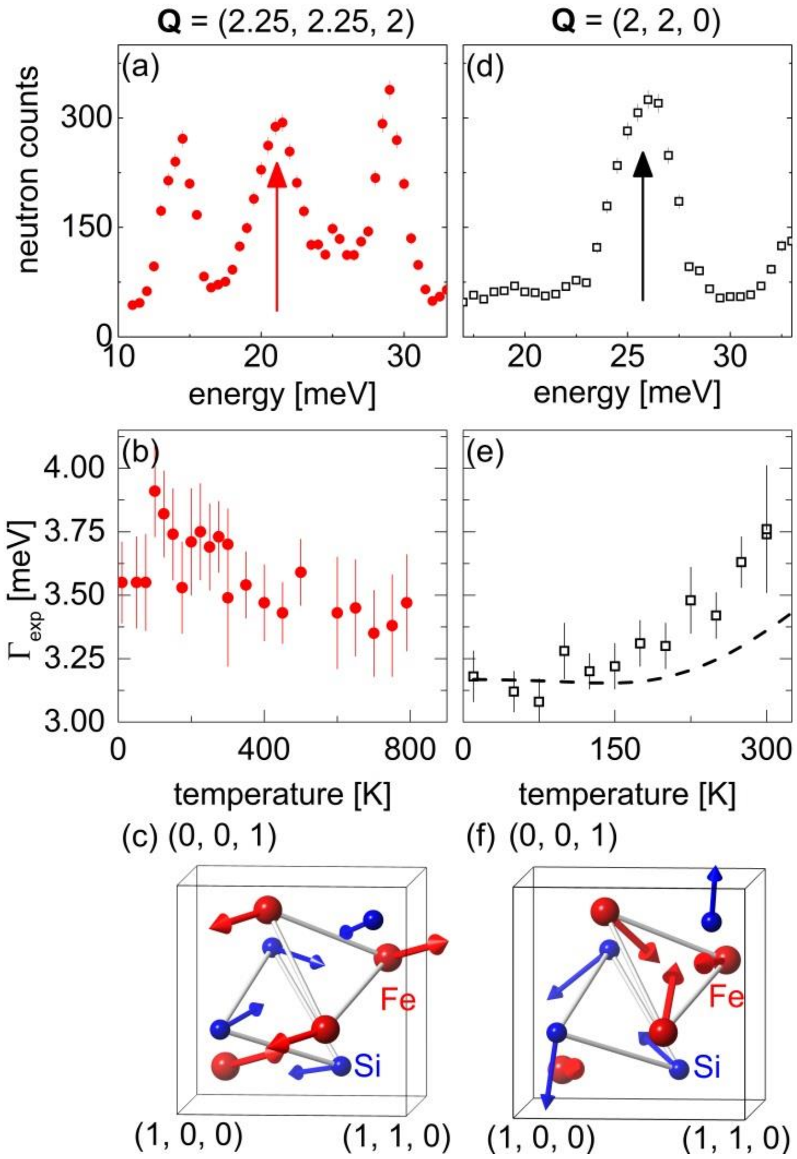

Supplementary Figure 5: Predicted phonon modes without and with strong temperature dependences. *(a),(d)* Raw data at T = 10 K, *(b),(e)* temperature dependence of the observed phonon line widths and *(c)(f)* calculated eigenvectors of the *(left side)* ΓM and *(right side)* Γ mode, which are indicated by the arrows in the top panels. The dashed line in *(e)* indicated the temperature dependent line width of the R_1 mode (shifted by 0.18 meV for comparison).

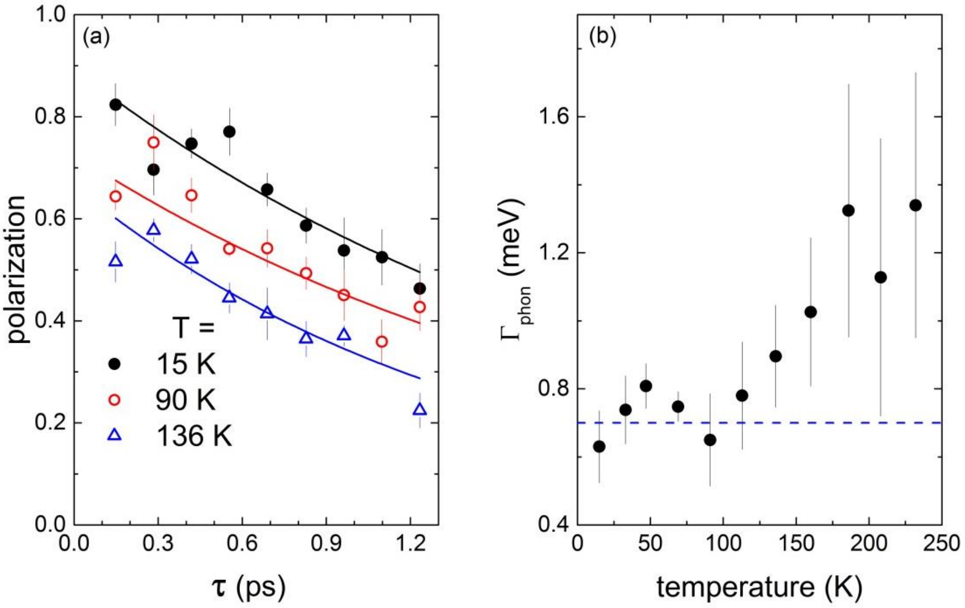

Supplementary Figure 6: TRISP data analysis of the intrinsic phonon line width of the R1 mode. *(a)* Shown are the observed polarizations over the spin echo time τ at three different temperatures. Lines are fits to the data of the form $P(\tau_{NSE}) = P_0 \exp\left(-\Gamma_{phon}\tau_{NSE}\right)$. (b) Results for Γ_{phon} (FWHM) at all investigated temperatures. The blue dashed line denotes the average value of the results for T ≤ 90K, which we assign to anharmonic contribution to the phonon line widths and were subtracted for the data shown in Fig. 3.

Supplementary Note 1

Polarized neutron scattering

The paramagnetic scattering in FeSi up to energy transfers of 80 meV was studied with polarized thermal neutrons on the TAS IN20 at ILL, Grenoble. Polarizing Heusler monochromator and analyzer have been used. The paramagnetic scattering intensities can be separated from other scattering processes by taking the difference of the measured spin-flip scattering for two different orientations of the guide field present at the sample position, i.e. horizontal field (HF) and vertical field (VF) [7],

$$\frac{1}{2}\left(\frac{d^2\sigma_{mag}}{d\omega\, d\Omega}\right)=\left(\frac{d^2\sigma^{(+,-)}}{d\omega\, d\Omega}\right)_{HF}-\left(\frac{d^2\sigma^{(+,-)}}{d\omega\, d\Omega}\right)_{VF}.$$

By setting the analyzer to a fixed final energy of 35 meV, energy transfers up to 80 meV could be achieved. In this study we focused on measuring the paramagnetic fluctuations close to reciprocal lattice vectors with large ferromagnetic structure factors, i.e. (1+ξ, 1+ ξ, 0+ ξ,), (1+ ξ, 1+ξ, 1+ ξ), (1+ ξ, 1+ξ, 2+ ξ) and $\xi = 0.075$. Magnetic scattering is strongest at small momentum transfers. However, we needed the larger wave vector values in order to close the scattering triangle at energies $E > 30$ meV. Hence, we performed the scans shown in Fig. 3(b) actually in scans at different wave vectors with different energy ranges. The obtained intensities were corrected for the ferromagnetic structure factors SF_{FM} of the reciprocal lattice points being $SF_{FM} = 1, 1.23$ and 0.47 for $\boldsymbol{\tau} = (1,1,0), (1,1,1)$ and $(1,1,2)$, respectively. Supplementary Figure 4(a) shows the corresponding three data sets for the measurement at $T = 300$ K.

The wave vector dependence of magnetic scattering in FeSi has been studied using polarized neutrons in the 1980s [4,5]. Here, we re-analyze the reported data in order to extract the experimentally observed magnetic correlation length ξ. The dynamic susceptibility reads $\chi(q,\omega) = \frac{\chi_0}{1+(q\xi)^2 - i\omega/\gamma}$, with χ_0 the static susceptibility. γ is a damping parameter and ξ denotes the correlation length. For small energy transfers, i.e., $\hbar\omega \ll k_B T$, the elastic scattering can be expressed as $\boldsymbol{S(q,0)} = \frac{\boldsymbol{k_B T}}{\boldsymbol{\hbar}} \cdot \frac{\boldsymbol{\gamma^{-1}\chi_0}}{\boldsymbol{(1+(q\xi)^2)^2}}$. Fitting this expression to published data on paramagnetic scattering at zero energy transfer[5] [Supplementary Figure 4(b)] we extract a magnetic correlation length in FeSi at T = 300 K of $\xi = (3.6 \pm 0.4)$ Å, i.e., 80% of the observed lattice parameter. The distance from an Fe ion to its 6 nearest Fe neighbors is 2.76 Å. Hence, on a local scale probed by the atomic movements within a phonon pattern, FeSi indeed can be regarded as a ferromagnet at elevated temperatures.

Supplementary Note 2

Ab-initio calculations based on density-functional-perturbation theory (DFPT)

Phonon energies in the quasi-harmonic approximation

A small softening of phonon energies with increasing temperature can be expected due to thermal expansion of the lattice. The Grüneisen parameter relates the strength of the softening with the change in the volume of the unit cell. If averaged over all energies and wave vectors, it acquires typically values of 1.5 – 2. In principle, however, the Grüneisen parameter depends on the energy and polarization of a phonon mode.

In order to have a mode-selective estimate of the phonon softening due to thermal expansion, we performed DFPT calculations with lattice constant values reported for temperatures T = 10 K, 100 K, 200 K, 300 K, 400 K, 500 K and 600 K[6]. Results plotted in Supplementary Figure 5(c) demonstrate that the expected relative softening due to thermal expansion is different for different modes. For instance, a high-energy mode at 54 meV at the **R** point shows a relative softening of only 3%, whereas the R1 mode energy is reduced by 6%. We note that the former is in good agreement with a 2% increase in the unit cell volume and a Grüneisen parameter of 1.5 as suggested in Ref. [8]. Obviously, this is different for the R1 mode.

This approach of using DFPT in order to estimate the phonon energies as function of temperature is known as the quasi-harmonic approximation and was validated in previous work[9-11].

Calculations for metallic FeSi

FeSi has an insulating ground state and our calculations of the electronic structure show indeed a strong suppression of electronic states at E_F, reminiscent of a gap in the excitation spectrum [Supplementary Figure 5(a)]. The charge gap was reported to close around room temperature[12] and, hence, a coupling of phonons to electronic states near E_F becomes possible.

DFPT is widely used to calculate the electronic contribution to the line widths of each phonon mode as function of energy and momentum in metallic compounds like charge-density-wave materials[13,14] or conventional superconductors above their respective transition temperatures[15,16].
DFPT calculations require a numerical smearing σ of the electronic bands due to the finite momentum mesh used. It was noted that this smearing can simulate a temperature effect[17-19]. Using $\sigma = 0.2$ eV in our calculations for FeSi effectively smears out all indications for a gap in the electronic excitation spectrum and yields a metallic like electronic density of states, eDOS [Supplementary Figure 5(a)]. The resulting metallic eDOS looks very similar to calculations by Jarlborg et al.[20] Based on this electronic structure it is possible to investigate the above discussed electron-phonon-coupling in the high-temperature, metallic phase of FeSi. We calculated the line widths of individual modes based on the electron-phonon coupling. Here, a denser 16x16x16 k-point mesh was employed for better convergence. Our calculations yield electronic contributions to the phonon line widths of no more than 0.1 meV for any mode in FeSi.

Spin polarized phonon calculations

Our experimental results suggest a close relationship between the increase in the phonon line widths of the R1 and R2 modes as a function of temperature with the temperature dependent magnetic moment in FeSi. The effect of a magnetic ground state on the lattice dynamical properties can be estimated employing spin polarized DFPT calculations. This has been done, e.g., for the Fe-based superconductors and related compounds[21,22] as well as for MnSi[23]. For the experimental lattice constant of FeSi, i.e. a = 4.48 Å, spin-polarized calculations show that a magnetic state is not stable.
However, we found that increasing the lattice constant to a* = 4.65 Å results in a stable magnetic ground state for FeSi. Using a larger lattice constant was further motivated by the presence of magnetic order in isostructural FeGe having a lattice constant of 4.7 Å.[24] FeGe features a helical magnetic order with an extremely long pitch of the helix of 700 Å. Further, it was shown that for FeGe a reduced lattice constant (e.g. by applying high pressures) leads to a non-magnetic semi-conducting ground state as it is found in FeSi[25].

The spin-polarized eDOS for spin-up and –down are shown in Supplementary Figure 5(b). The ordered magnetic moment is $0.8\mu_B$ per Fe atom. Optimizing the internal structure of the unit cell for the latter value for the lattice constant, we are able to perform magnetic and non-magnetic calculations and, thus, infer the effect of magnetism on the phonon energies of FeSi. One has to be aware of the fact that the spin-polarized calculation assumes a magnetically ordered state, which is not the case in FeSi at any temperature. However, short-range ferromagnetic correlations between the Fe spins have been discussed in the literature[26].

Supplementary Note 3

ΓM_1 and Γ mode – temperature dependences

Spin-polarized calculations predict strong EPC for many phonon modes in FeSi. In the main text we focus on the longitudinal modes at the R point, R1 and R2. Apart from this high symmetry point, Supplementary Figure 8 shows measurements at two other wave vectors, at which we could accurately determine the experimental background and, thereby, were able to extract the phonon line widths as a function of temperature. Two wave vectors are particularly interesting, as DFPT predicts $\Gamma_{phon} \approx 0$ for the mode at **q** = (0.25, 0.25, 0) and E = 21 meV [arrow in Supplementary Figure 8(a)], whereas the mode in the zone centre at E = 26 meV [arrow in Supplementary Figure 8(d)] should show a line width even larger than that of the R1 and R2 modes. For simplicity, we call them ΓM and Γ modes. Panels (b) and (e) show the observed phonon line widths for both phonons. In agreement with DFPT we see no measureable increase of Γ_{exp} for the ΓM mode. The Γ mode, however, clearly features an increasing Γ_{exp} already below room temperature. For comparison, the temperature dependent $\Gamma_{exp}(T)$ of the R1 mode (shifted for the slightly worse calculated resolution for the Γ mode) is indicated by the dashed line in panel (e). Obviously, $\Gamma_{exp}(T)$ of the zone centre mode increases already at low temperatures and shows a larger line width at room temperature than the R1 mode.
As we discuss in the following in detail, we found that strong EPC apparently goes hand in hand with a particular type of lattice vibrations, in which the Fe-Fe distances are affected by the atomic movements. In order to understand the presence or absence of strongly increased phonon line widths we computed the eigenvectors of the R1 and R2 modes [Fig. 4(c),(d)] as well as the patterns for the Γ and ΓM modes [Figs. S8(c),(f)]. Looking at one unit cell of FeSi, i.e. 4 Fe and 4 Si ions, we found the following characteristics: For the R1 and R2 modes, the Fe ion located at (u, u, u), u = 0.127 r.l.u., within the unit cell has a displacement nearly parallel to the [111] direction which strongly changes the distance to the other three Fe atoms, which together make roughly a rotational movement around the [111] axis of the unit cell. The pattern of the Γ mode features

a pattern with even stronger changes of the inter-Fe distances. Whereas the Fe ion at (u, u, u), u = 0.127 r.l.u., makes a slightly less direct movement towards the other three Fe ions, the latter themselves exhibit a breathing-type pattern, i.e., the three ions move simultaneously towards the center of a circle defined by their equilibrium positions [Supplementary Figure 8(f)]. On the other hand, the ΓM mode exhibits evasive movements of the Fe ions, which do not change the interatomic distances very much [Supplementary Figure 8(c)]. The analysis of the eigenvectors indicates that phonons having patterns with strongly renormalized Fe-Fe distances are affected by additional damping going beyond the quasiharmonic approximation. The effect grows with the temperature-induced paramagnetic moment. Hence, we propose that a direct coupling between the fluctuating electric polarization of the ionic movements and the magnetic moments is responsible for the strong phonon renormalization. FeSi is unique in that it offers to study this effect in a crossover from a non-magnetic insulator towards a nearly ferromagnetic metal, i.e., spanning the magnetic moment range from zero to more than $2\mu_B$.

Supplementary Note 4

Neutron spin echo measurements

The intrinsic phonon line widths Γ_{phon} of the R_1 mode at 15 K ≤ T ≤ 225 K were investigated using the three axis spin echo spectrometer TRISP at the neutron research source Hans-Maier Leibnitz (FRM II) in Garching. This instrument applies the neutron spin echo (NSE) technique and is able to determine line widths of the order of a few μeV [27,28]. A bent neutron guide provides a polarized incident neutron beam. The incident energy is selected by a double-focusing graphite monochromator while a velocity selector in front of the monochromator suppresses higher-order energies. Typically, radio-frequency (RF) coils before and after the sample provide the magnetic fields for the neutron precession. Due to the relatively large anharmonic line width of the R_1 mode already at very low temperature, we exchanged the RF coils for DC coils, which enabled us to measure at lower spin-echo times, i.e., $\tau < 2$ ps. We used a fixed final energy of 18.6 meV ($|\mathbf{k}_f| = 3.0$ Å^{-1}) selected by a Heusler analyzer.

Supplementary Figure 3(a) shows the measured polarization for various spin-echo times τ at three selected temperatures. Lines are fits to the data of the form $P(\tau_{\mathrm{NSE}}) = P_0 \exp(-\Gamma_{\mathrm{phon}}\tau_{\mathrm{NSE}})$, where Γ_{phon} is the line width of the Lorentzian line shape describing the intrinsic phonon excitation. Results for Γ_{phon} are shown in Supplementary Figure 3(b). In order to extract the temperature dependent part of the line width [Fig. 3(a)], we subtracted the average value of the determined line width for $T \leq 90$ K, $\Gamma_{\mathrm{av}} = 0.7$ meV (blue dashed line). This contribution to the phonon line width originates most likely from anharmonic effects.

Supplementary References

1 Wertheim, G. K. *et al.* Unusual electronic properties of FeSi. *Phys Lett* **18**, 89-90 (1965).

2 Jaccarino, V., Wertheim, G. K., Wernick, J. H., Walker, L. R. & Arajs, S. Paramagnetic Excited State of FeSi. *Physical Review* **160**, 476-482 (1967).

3 Wolfe, R., Wernick, J. H. & Haszko, S. E. Thermoelectric properties of FeSi. *Phys Lett* **19**, 449-450 (1965).

4 Shirane, G., Fischer, J. E., Endoh, Y. & Tajima, K. Temperature-induced magnetism in FeSi. *Physical Review Letters* **59**, 351-354 (1987).

5 Tajima, K., Endoh, Y., Fischer, J. E. & Shirane, G. Spin fluctuations in the temperature-induced paramagnet FeSi. *Physical Review B* **38**, 6954-6960 (1988).

6 Vočadlo, L., Knight, K. S., Price, G. D. & Wood, I. G. Thermal expansion and crystal structure of FeSi between 4 and 1173 K determined by time-of-flight neutron powder diffraction. *Physics and Chemistry of Minerals* **29**, 132-139 (2002).

7 Shirane, G., Shapiro, S. & Tranquada, J. *Neutron Scattering with a Triple-Axis Spectrometer*. (Cambridge University Press, 2002).

8 Delaire, O. *et al.* Phonon softening and metallization of a narrow-gap semiconductor by thermal disorder. *P Natl Acad Sci USA* **108**, 4725-4730 (2011).

9 Baroni, S., Giannozzi, P. & Isaev, E. Density-Functional Perturbation Theory for Quasi-Harmonic Calculations. *Rev Mineral Geochem* **71**, 39-57 (2010).

10 Debernardi, A., Alouani, M. & Dreyssé, H. Ab initio thermodynamics of metals: Al and W. *Physical Review B* **63**, 064305 (2001).

11 Quong, A. A. & Liu, A. Y. First-principles calculations of the thermal expansion of metals. *Physical Review B* **56**, 7767-7770 (1997).

12 Menzel, D. *et al.* Electron-phonon interaction and spectral weight transfer in $Fe_{1-x}Co_xSi$. *Physical Review B* **79**, 165111 (2009).

13 Weber, F. *et al.* Optical phonons and the soft mode in 2H-$NbSe_2$. *Physical Review B* **87**, 245111 (2013).

14 Calandra, M. & Mauri, F. Charge-Density Wave and Superconducting Dome in $TiSe_2$ from Electron-Phonon Interaction. *Physical Review Letters* **106**, 196406 (2011).

15 Weber, F. *et al.* Electron-Phonon Coupling in the Conventional Superconductor YNi_2B_2C at High Phonon Energies Studied by Time-of-Flight Neutron Spectroscopy. *Physical Review Letters* **109**, 057001 (2012).

16 Heid, R., Bohnen, K.-P. & Renker. Electron-phonon coupling and superconductivity in MgB_2 and related diborides. *Adv. Solid State Phys.* **42**, 293 (2002).

17 Bohnen, K., Heid, R., Liu, H. J. & Chan, C. T. Lattice Dynamics and Electron-Phonon Interaction in (3,3) Carbon Nanotubes. *Physical Review Letters* **93**, 245501 (2004).

18 Weber, F. *et al.* Extended Phonon Collapse and the Origin of the Charge-Density Wave in 2H-$NbSe_2$. *Physical Review Letters* **107**, 107403 (2011).

19 Weber, F. *et al.* Electron-Phonon Coupling and the Soft Phonon Mode in $TiSe_2$. *Physical Review Letters* **107**, 266401 (2011).

20 Jarlborg, T. Electronic structure and properties of pure and doped ε-FeSi from ab initio local-density theory. *Physical Review B* **59**, 15002-15012 (1999).

21 Reznik, D. *et al.* Phonons in doped and undoped $BaFe_2As_2$ investigated by inelastic x-ray scattering. *Physical Review B* **80**, 214534 (2009).

22 Boeri, L., Calandra, M., Mazin, I. I., Dolgov, O. V. & Mauri, F. Effects of magnetism and doping on the electron-phonon coupling in $BaFe_2$ As_2. *Physical Review B* **82**, 020506 (2010).

23 Lamago, D. *et al.* Lattice dynamics in the itinerant helical magnet MnSi. *Physical Review B* **82**, 144307 (2010).

24 Lebech, B., Bernhard, J. & Freltoft, T. Magnetic structures of cubic FeGe studied by small-angle neutron scattering. *Journal of Physics: Condensed Matter* **1**, 6105-6122 (1989).
25 Yamada, A., Terao, K., Ohta, H. & Kulatov, E. Electronic structure and magnetism of FeGe with B20-type structure. *Physica B* **329-333**, 1131-1133 (2003).
26 Imada, M., Fujimori, A. & Tokura, Y. Metal-insulator transitions. *Review of Modern Physics* **70**, 1039-1263 (1998).
27 Keller, T. *et al.* Momentum-Resolved Electron-Phonon Interaction in Lead Determined by Neutron Resonance Spin-Echo Spectroscopy. *Physical Review Letters* **96**, 225501 (2006).
28 Aynajian, P. *et al.* Energy Gaps and Kohn Anomalies in Elemental Superconductors. *Science* **319**, 1509-1512 (2008).
